# Protective effects and therapeutic applications of ellagic acid against natural and synthetic toxicants: A review article

**DOI:** 10.22038/IJBMS.2022.64790.14267

**Published:** 2022-12

**Authors:** Karim Naraki, Maryam Rameshrad, Hossein Hosseinzadeh

**Affiliations:** 1 Department of Pharmacodynamics and Toxicology, School of Pharmacy, Mashhad University of Medical Sciences, Mashhad, Iran; 2 Student Research Committee, Mashhad University of Medical Science, Mashhad, Iran; 3 Natural Products and Medicinal Plants Research Center, North Khorasan University of Medical Sciences, Bojnurd, Iran; 4 Pharmaceutical Research Center, Pharmaceutical Technology Institute, Mashhad University of Medical Sciences, Mashhad, Iran

**Keywords:** Antidote, Anti-oxidant, Chemical toxic agent, Ellagic acid, Natural toxin, Protective

## Abstract

Traditional herbal drugs are widely used for the treatment of various diseases. Ellagic acid (EA) as an herbal polyphenol metabolite exists in many medicinal plants. EA has an important role against natural and chemical toxicities due to its antioxidant and anti-inflammatory properties. For this review, several search engines or databases such as PubMed, Scopus, the Web of Science, and Google Scholar were used, and the most relevant published papers till February 2022 were included.

The protective effects of EA against natural and chemical compounds are mediated through molecular mechanisms including scavenging of free radicals, modulation of proinflammatory cytokine synthesis, and reduction of lipid peroxidation. These properties make EA a highly fascinating compound that may contribute to different aspects of health; whereas, more studies are needed, especially on the pharmacokinetic profile of EA.

In this review, we selected articles that include the protective effect of EA against several synthetic and natural toxins such as aflatoxin, lipopolysaccharide, acrylamide, and rotenone.

## Introduction

**Figure 1:**
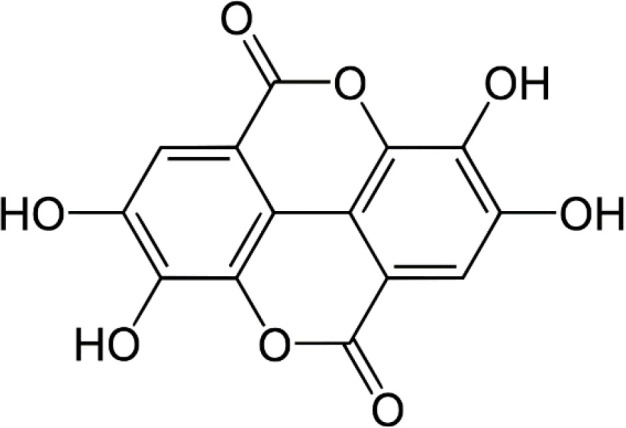
Chemical structure of ellagic acid

**Figure 2:**
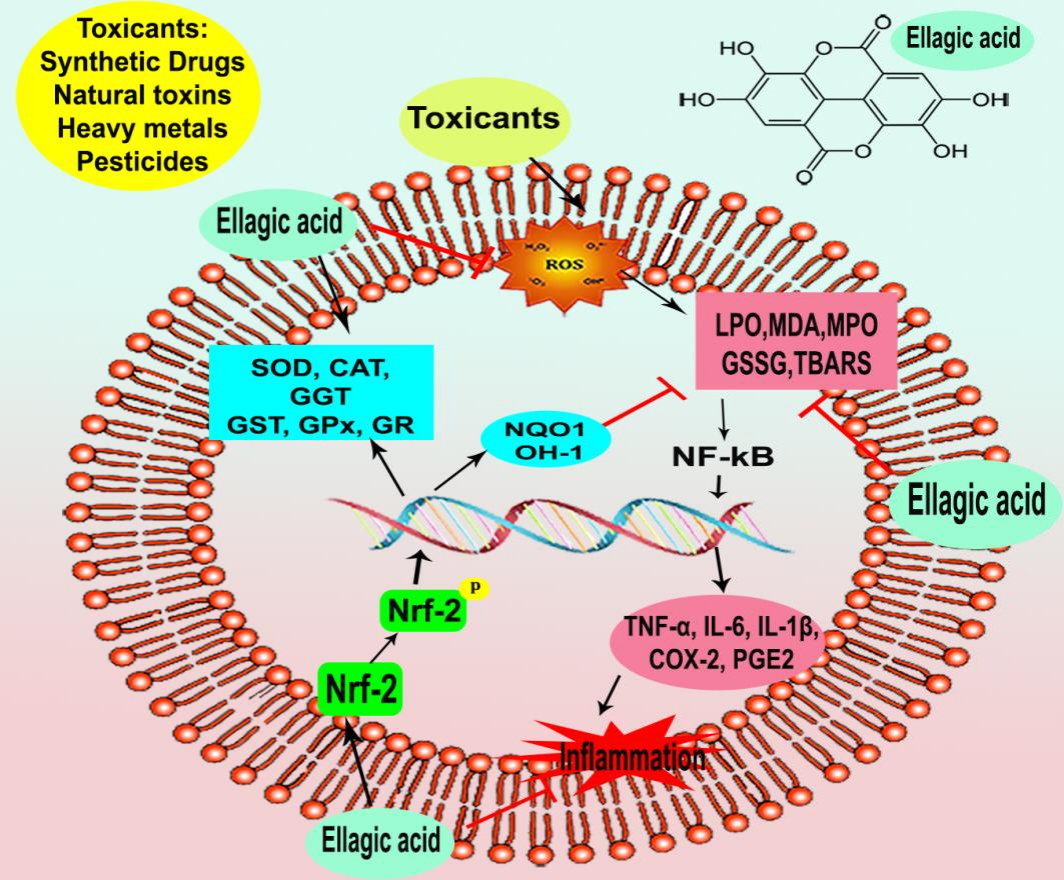
Schematic diagram that explains ellagic acid’s anti-inflammatory and antioxidant properties against chemical agents and natural toxins

CAT: catalase; COX-2: cyclooxygenase 2; GGT: gamma-glutamyltransferase; GPx: glutathione peroxidase; GR: Glutathione reductase; GSSG: Glutathione disulfide; GST: glutathione-S-transferase, IL-6: interleukin-6; IL-1β: interleukin-1β; LPO: lipid peroxidation; MDA: malondialdehyde; MPO: myeloperoxidase; NF-κB: nuclear factor kappa-B; NQO1: NAD(P)H quinone oxidoreductase; Nrf2: nuclear erythroid 2-related factor 2; PGE2: prostaglandin-E2; SOD: superoxide dismutase; TBARS: thiobarbituric acid reactive substances; TNF-α: tumor necrosis factor-alpha

Ellagic acid (EA) (2,3,7,8-tetrahydroxy [1]-benzopyranol [5,4,3-*cde*] benzopyran-5,10-dione) C_14_H_6_O_8_; (Figure 1) belongs to the class of polyphenol extractives (tannins) that is isolated from many fruits, nutgalls, and plants such as raspberries, strawberries, grapes, pomegranate, black currants, longan seeds, and green tea (1). EA usually is conjugated with a glycoside moiety such as glucose, rhamnose, arabinose, or bounded in the form of ellagitannins that have astringent properties and act as a defense system against microbial and animal attacks (2). Free EA is commonly formed during food processing or in physiological conditions in the human gastrointestinal tract (3). EA, with a molecular mass of 302.197 g/mol, is an extremely thermostable molecule with low water solubility and moderate solubility in alcohol (4). Previous investigations have reported numerous pharmacological activities of EA such as antioxidant, anti-inflammatory, neuroprotective, nephroprotective, and hepatoprotective properties (5-8). The suggested mechanisms for EA protective effects are *via* activation of antioxidant enzymes including superoxide dismutase (SOD), catalase (CAT), glutathione peroxidase (GPx), and glutathione-*S*-transferase (GST), and also modulation of various signaling pathways such as nuclear erythroid 2-related factor 2 (Nrf2), phosphoinositide 3-kinase and glycogen synthase kinase 3 beta (GSK-3β) (6, 9). Furthermore, it suppresses pro-inflammatory markers such as cyclooxygenase (COX-2) and nuclear factor-kappa B (NF-кB) (10-12). It has been indicated that EA is an efficient radical scavenger of OH^•^, methoxyl (OCH_3_^•^), and nitrogen dioxide (NO_2_^•^) in the body organs due to its antioxidant effects (13, 14). Previous *in vivo* and *in vitro* studies have indicated the ameliorative effects of EA against several malignancies including colorectal, breast, and prostate cancers, and also leukemia, lymphoma, and melanoma (10, 15). In this review, we aim to explain the antioxidant, anti-inflammatory, and ameliorative properties of EA against various types of toxins or toxic agents including pharmaceuticals, heavy metals, and pesticides (Figure 2).

***Method***

For this review, we used several search engines or databases such as PubMed, Scopus, the Web of Science, and Google Scholar, and we selected the most relevant published papers till February 2022. The selected keywords were “ellagic acid”, “antioxidant”, “anti-inflammatory”, “protective”, “ameliorative”, “hepatoprotective”, “neuroprotective”, “nephroprotective”, and “cardioprotective”. Only articles written in English and published in peer-reviewed scientific journals were selected.

***Protective effects of ellagic acid against adverse effects or toxicity of synthetic drugs***

*Cyclophosphamide*

Cyclophosphamide (CPM) is an alkylating antineoplastic agent that is commonly administered for the treatment of various disorders including leukemias, myeloblastoma, breast cancer, malignant lymphomas, and ovarian carcinoma (16). CPM has a wide range of adverse effects, including nephrotoxicity, genotoxicity, and reproductive toxicity (17). EA administration (10 mg/kg, intraperitoneal* (*IP*)*) to rats significantly decreased liver damage indicator enzymes such as aspartate aminotransferase (AST), alanine aminotransferase (ALT), alkaline phosphatase (ALP), and gamma-glutamyltransferase (18). The antioxidant effect of EA against CPM-induced oxidative stress was manifested via the decrease in malondialdehyde (MDA), advanced oxidized proteins product contents, xanthine oxidase (XO) activities, elevated glutathione (GSH) concentrations, and CAT activity. Furthermore, EA treatment increased the B-cell lymphoma 2 (Bcl-2)/ Bcl-2-associated X protein (Bax) ratio and diminished the expression of CD15, involved in the extracellular adhesion and migration of cells, in the hepatocytes and lymphocytes which was induced via CPM (18). CPM-induced testicular and spermatozoa toxicity was reported to be associated with oxidative stress and apoptosis, a decrease in epididymal sperm content and motility, and significantly increased MDA levels in male rats. EA (2 mg/kg, PO) administration protected rats against the development of these detrimental effects, significantly increased the number of Bcl-2-positive cells, reduced the number of Bax-negative cells, and ameliorated the CPM-induced immunohistochemical damage (19). Treating rats with EA (2 mg/kg, PO) protects against CPM-induced lipid peroxidation and structural damages in spermatozoa and testicular tissue of rats (20). Administration of CPM to mice increased MDA and XO levels with depletion in GSH content, decreased antioxidant enzyme activities including GPx, glutathione reductase (GR), CAT, and quinone reductase, and induced DNA strand breaks in kidney tissue. EA supplementation in mice (100 mg/kg, PO) significantly suppressed the kidney serum toxicity markers including blood urea nitrogen, creatinine, and lactate dehydrogenase (LDH) which were induced by CPM (21). Moreover, EA (15 mg/kg, IP) treatment modulated enhancement of hydroxyproline level, lipid peroxidation (LPO), myeloperoxidase activity, nitric oxide (NO), and protein carbonyl generation in lungs of rats exposed to CPM. EA treatment restored the activity of antioxidant enzymes such as GPx and GR and reduced reactive oxygen species (ROS) generation in the lung tissue of rats exposed to CPM (22) (Table 1).

*Cyclosporine A*

Cyclosporine A (CsA) is a common immunosuppressive agent that is widely used in candidates for solid-organ transplantation (23). Besides the beneficial effect of CsA, several related toxicities have been reported including reproductive toxicity, nephrotoxicity, hepatotoxicity, and cardiotoxicity through oxidative stress, autophagy, and depletion of antioxidant enzymes (23). In rats, CsA diminished the weights of testes and ventral prostate, epididymal sperm level, sperm motility, GSH, and CAT, and increased MDA level in testicular tissue, while EA (10 mg/kg, PO) treatment attenuated all the CsA-induced negative changes (24). Also, EA significantly ameliorated CsA-induced germinal cell necrosis, interstitial edema, spermatogenic arrest, and capillary congestion (24). EA treatment (10 mg/kg, PO) significantly increased the GSH level, GPx, and CAT activities and decreased the MDA content of the kidney, liver, and heart in rats exposed to CsA. Furthermore, CsA caused severe damage in the kidney, liver, and heart tissues which were noticeably ameliorated by EA supplementation (25). EA (10 mg/kg, subcutaneous* (*SC*)*) co-treatment with CsA improved CsA toxic side effects in testis and bone marrow tissues with a significant reduction in oxidative stress, LPO, and MDA levels. EA therapy elevated CAT and peroxidase activities and GSH concentration (26). CsA induced liver damage by increasing the serum hepatic enzymes such as AST, ALT, ALP, and LDH and elevating LPO markers such as thiobarbituric acid reactive substances (TBARS) and hydroperoxides. Administrations of EA (50 mg/kg, PO) in rats significantly decreased the activities of hepatic marker enzymes and also reduced the levels of TBARS and hydroperoxides (27) (Table 1).

*Doxorubicin*

Doxorubicin (Dox) is a valuable anticancer drug widely used in the treatment of various hematological and solid tumor malignancies. Although DOX is a very useful drug, its therapeutic use has been limited because of its acute and chronic cardiac toxicities and neurotoxicity (28). Exposure of rats to Dox declined testicular weight, testicular glycogen, sperm count and motility, and serum testosterone level. It increased the histopathological changes, oxidative stress, and TNF-α level, and administration of EA (10 mg/kg, PO) reversed these changes (29). Supplementation of Dox-treated mice with EA (1 g, PO) reduced ROS, interleukin-6 (IL-6), and IL-10 levels. Also, it decreased MDA and XO activity and monocyte chemoattractant protein-1 and TNF-α levels, and it enhanced GSH content and GPx, SOD, and CAT activity in heart tissue (30). Furthermore, Dox exposure increased caspase-3, nuclear factor kappa-B (NF-κB), p50, and p65 protein content, and EA administration down-regulated these inflammatory and apoptotic proteins (30). A previous study indicated that DOX elevated MDA, TNF-𝛼, inducible nitric oxide synthase (iNOS), caspase-3, and cholinesterase levels and significantly reduced GSH levels, monoamines including serotonin, dopamine, and norepinephrine in the brain; however, EA (10 mg/kg, PO) effectively protected rats against DOX-induced neurotoxicity by its antioxidant, anti-inflammatory, and antiapoptotic properties (31) (Table 1).

*Gentamicin*

Gentamicin is an aminoglycoside antibiotic discovered in 1963 and widely used in clinics against gram-negative bacillary infections especially those originating from* Pseudomonas aeruginosa*. Gentamicin accumulation in several organs induced toxicity such as nephrotoxicity and ototoxicity (32). EA (10 mg/kg, PO) co-treatment with gentamicin in rats significantly ameliorated oxidative stress markers including LPO and MDA, and increased CAT, SOD enzyme activity, and GSH content in the kidney tissue (33). Furthermore, they reported that EA administration increased the Bcl-2/Bax ratio and prevented mitochondrial membrane potential (MMP) loss in kidney tissue against gentamicin (33) (Table 1).

*Isoproterenol*

Isoproterenol (ISP), a synthetic β-adrenoceptor agonist, is commonly used in the treatment of cardiac dysfunction such as bradycardia, thioridazine-induced torsade de pointes, and heart block (34). ISP induces myocardial infarction (MI) through alteration in biochemical and histological changes, oxidative stress, LPO, and pro-inflammatory cytokines (35). EA administration (15 mg/kg, PO) in ISP-treated rats noticeably decreased levels of cardiac markers such as cardiac troponin-I, creatine kinase, LDH, C-reactive protein, TBARS, and lipid hydroperoxides (36). Also, EA displayed high potential activity to elevate the anti-peroxidative enzymes including SOD, GST, and CAT, and also restored the GSH content in ISP-induced myocardial infarction (36) (Table 1).

*Methotrexate*

Methotrexate (MTX) as an antimetabolite agent for cancer treatment could induce many adverse effects including renal dysfunction, myelosuppression, mucositis, hepatotoxicity, and in severe cases multiorgan failure (37). Administration of MTX in rats induced oxidative stress, increased MDA levels, Bax/Bcl-2 ratio, cytochrome-c release, and caspase-3/9 level, and decreased mitochondrial outer membrane potential; furthermore, it elevated the concentration of pro-inflammatory factors such as NF-ĸB and IL-6 that were decreased by EA (10 mg/kg, PO) administration. EA therapy considerably up-regulated both Nrf2 and HO-1 which were down-regulated in MTX-treated rats (38). EA (10 mg/kg, PO) treatment in rats significantly reduced MDA, NO, and prostaglandin-E2 levels and diminished the activity of myeloperoxidase, XO, and AD, and also increased GSH content level in the intestinal tissue of rats treated with MTX; moreover, EA administration protected the intestinal tissue against histopathological changes caused by MTX (39). Moreover, EA (10 mg/kg, PO) protected the liver against MTX-induced damage through its antioxidant properties by reducing the serum liver enzymes, including AST, ALT, and ALP (40). Also, EA treatment prevented the elevation of MDA and LPO by activating the antioxidant enzymes such as SOD, CAT, and GST, and also restoring the GSH content level in the liver of rats exposed to MTX (40) (Table 1).

*Paracetamol*

Paracetamol (acetaminophen) is the most used analgesic and antipyretic medicine around the world (41). Chronic administration of paracetamol could induce several toxicities including hepatotoxicity, cardiotoxicity, nephrotoxicity, and neurotoxicity (41). Paracetamol in rats induced hepatic damage by increasing the activities of marker enzymes (ALT, AST, and ALP) in serum and MDA levels in the liver, and also reducing CAT and SOD activity and GSH level; however, EA (100 mg/kg, PO) administration prevented the liver damage induced with paracetamol (42) (Table 1).

*Sodium valproate*

Sodium valproate is a widely used antiepileptic drug with a wide-ranging activity and mechanism of action. Sodium valproate is a well-tolerated medicine in the patient, but it causes several health problems including hepatotoxicity and hyperammonemia encephalopathy (43). Sodium valproate produced reproductive toxicity and associated histological changes in male rats through the significant suppression of sperm count and motility which ameliorated via EA (50 mg/kg, PO) treatment (44). Furthermore, EA co-treatment with sodium valproate protected the male rats’ reproductive system against necrosis, seminiferous tubule atrophia, formation of the multinucleated giant cell, interstitial edema with congestion, reduction in germinal cell count, and compromised spermatogenesis (44) (Table 1).

*Streptozocin*

Streptozocin or streptozotocin is a glucosamine-nitrous-urea compound that first was introduced for treating metastatic pancreatic islet cell cancer, and in experimental models, it is used to induce insulin-dependent diabetes mellitus, non-insulin-dependent diabetes mellitus, and Alzheimer’s disease (AD) (45). EA administration (35 mg/kg, PO) to rats that received intracerebroventricular streptozotocin prevented them from the loss of cognitive abilities; it decreased the content of TBARS and elevated the level of GSH, SOD, and CAT activity in the brain tissue. Furthermore, EA administration decreased acetylcholinesterase and LDH activity, and TNF-α and eNOS levels in the brain (46). A similar study suggests that EA (50 mg/kg, PO) supplementation in rats treated with streptozocin significantly reduced MDA content, total oxidant status, oxidative stress index, and NO levels, and reverses levels of total antioxidant status, CAT, and paraoxonase activities in the brain tissue (47) (Table 1).

***The protective effects of ellagic acid against natural toxins***

Natural toxins usually are poisonous secondary metabolites that are produced by living organisms, which are typically not harmful to the organisms themselves but can impact human or animal health when consumed. Some plant-based foods may also be toxic if they are not processed or cooked appropriately before consumption. Besides, some toxins have been reported as valuable molecules for drug development.

*Aflatoxin B1*

Aflatoxins belong to mycotoxins and are produced by *Aspergillus flavus* and *Aspergillus parasiticus*. They are found in spoiled foodstuff, milk, meat, and eggs of animals who were fed aflatoxin-contaminated foods. Among them, AFB1 is the most hazardous type (48). Its exposure can cause nausea, vomiting, abdominal pain, and convulsion, and its chronic exposure can also lead to several health disorders like hepatotoxicity, immunotoxicity, and teratogenicity (49). EA (300 mg/kg, IP) prevented AFB1 genotoxicity in the bone marrow and lung cells (50). Treating *Salmonella* with AFB1 leads to mutagenicity and EA pretreatment diminished it by building an extracellular complex with AFB1 and reducing the interaction of AFB1 with the bacteria (51) (Table 2).

*Concanavalin A*

Concanavalin A (ConA), a Ca^2+/^Mn^2+^-dependent and mannose/glucose-binding lectin from Jack bean seeds, has a potent anti-cancer effect (52). ConA induced hepatitis by activating toll-like receptor (TLR) signaling pathways (52). EA (200 mg/kg, PO) pretreatment diminished the plasma levels of aminotransferases (ALT and AST) and liver necrosis in ConA-induced hepatitis (53). Also, EA significantly decreased TLR2 and 4 protein and mRNA expression in liver tissue. Also, EA supplementation decreased the expression of NF-κB and the expression of proinflammatory cytokines including TNF-α, IL-6, and interleukin-1β (IL-1β) in ConA-treated mice (53) (Table 2).

*Glutamate*

Glutamate, a non-essential amino acid, is a principal excitatory neurotransmitter in the brain. Animal experimental studies have shown it has the potential to induce testicular and neural toxicity (54). Monosodium glutamate which is used as a food additive in commercial foods could be considered an important source of exposure to the high level of glutamate (55), although, at the physiological level, the blood-brain barrier may restrict the passage of glutamate from the blood into the brain (56). Glutamate exposure in rats significantly reduced testicular antioxidant biomarkers including SOD, GPx, GRx, and CAT, and increased myeloperoxidase and XO activity. EA (20 mg/kg, PO) elevated testosterone hormone content, improved histological and ultrastructure testicular damages, and inhibited the redox state in male rats (57) (Table 2).

*Lipopolysaccharide*

Lipopolysaccharide (LPS) is the main component of the outer membrane of Gram-negative bacteria (58). It is the major reason for a severe systemic inflammatory response syndrome that is called sepsis (59). In the liver, it induced the activation of macrophages, the NF-κB pathway, and the production of inflammatory mediators including TNF-α and IL-6 which lead to hepatic failure (60). EA (20 mg/kg, PO) supplementation in mice exposed to LPS reduced hepatic MDA content, TNF-α, and serum ALT and AST levels. The inhibition of NF-κB with EA was found to be related to an increase in the activation of Nrf2/HO-1 signaling pathways in the liver tissue (60) (Table 2).

*Ethanol*

Ethanol toxicity can occur by ingestion of a large quantity of ethanol in the form of beverage ethanol, mouthwash, cologne, and cough medicine (61). Ethanol abuse is one of the most important world health issues that is associated with organ dysfunction and diseases such as hepatitis, liver cirrhosis, cardiomyopathy, and brain disorders (62). The chronic consumption of ethanol leads to inflammation in the liver tissue by enhancing the production of inflammatory mediators such as transforming growth factor-β1, TNF-α, and ROS which contribute to hepatocyte dysfunction, necrosis, apoptosis, and fibrosis (63, 64). EA (90 mg/kg, daily for 45 days, PO) co-administration in rats with ethanol (7.9 g/kg, daily for 45 days, PO) meaningfully improved the status of antioxidants and decreased TBARS, hydroperoxides, NO, protein carbonyl content, and liver marker enzymes including ALT, AST, LHD, and ALP, and also, enhanced GSH level and SOD activity in the liver (65). EA (90 mg/kg, PO) supplementation to rats inhibited alcohol-induced toxicity by improving body weight, restoring antioxidant status (SOD, CAT and GPx, and GSH level), and reduction of lipid content in the circulation; furthermore, EA diminished liver marker enzymes including gamma-glutamyltransferase, AST, ALT, and ALP (65). Ethanol-induced toxicity in HepG2 cells is accompanied by elevation of NO and transforming growth factor-beta production, which EA (100 μM) administration regulated NO and transforming growth factor-beta production in ethanol-stimulated HepG2 cells (66) (Table 2).

*Nicotine*

Nicotine with a 1-2 hr half-life is the primary component of tobacco. It is highly addictive with liability for widespread abuse. It is metabolized primarily in the liver and then excreted by the kidneys (67). Nicotine causes uterine vasoconstriction and placenta damage by enhancing the MDA level and LPO (68). Nicotine treatment in rats significantly decreased body weight, total GSH, and SOD activities, and increased MDA and NO levels in the kidney tissue which are reversed with EA (60 mg/kg, IP) co-treatment (69) (Table 2).

***Protective effects of ellagic acid against the toxicity of pesticides***

*Malathion*

Malathion (MAL), a known synthetic organophosphate pesticide, is widely used in agriculture, industry, and veterinary medicine (70). Chronic MAL exposure is associated with several organ toxicities such as neurotoxicity, cardiotoxicity, hepatotoxicity, and immunotoxicity in both humans and animals (71). Exposure to fish with MAL caused oxidative stress, significantly increased MDA, and diminished the activities of SOD and CAT in the liver, kidney, and gills which were reversed by EA administration (72) (Table 3).

*Paraquat*

Paraquat (PQ) is an efficient and widely used herbicide for destroying weeds that may decrease crop yields, however, PQ is highly toxic through generation of ROS, LPO, and oxidation of GSH to glutathione disulfide (73). The protective effects of EA (85 mg/kg, PO) against PQ-induced nephrotoxicity were shown to be mediated via improving tissue structure and increasing the total antioxidant status (74). The cell viability was decreased and ROS, LPO, and LDH increased in the A549 cell line by PQ treatment, and these effects were reversed by EA pretreatment. Moreover, EA (80 μM) significantly up-regulated the level of Nrf2, HO-1, and NAD(P)H quinone oxidoreductase that protects the A549 cell line against PQ toxicity (75) (Table 3).

*Phosalone*

Phosalone is an organophosphate pesticide that is widely used around the world. Phosalone induced several toxicities in organs including the liver, brain, kidney, and heart by enhancing ROS and LPO which leads to DNA fragmentation (76). Phosalone in rat embryonic fibroblast (REF) cells decreased cell viability and increased oxidative stress markers (ROS and LPO) and inflammatory cytokines (TNF-α, IL-1β, IL-6, and NF-κB) (77). However, EA (100 nM) pretreatment protected cells by suppressing free radicals and ROS formation, decreasing the expression and protein levels of p38 and p53, and reduction of inflammatory factors including TNF-α, IL-1β, IL-6, and NF-κB (77). Induction of NF-κB leads to p53 activation which causes cell arrest in G1/S (78) (Table 3).

*Rotenone*

Rotenone, a known widely used insecticide, is a potent mitochondrial complex I inhibitor that induces neurotoxicity and causes neurodegenerative disorders such as Parkinson’s disease (79). EA (100 mg/kg, PO) supplementation in mice protected them against rotenone neurotoxicity via activation of the Nrf2/HO-1 signaling pathway. Nrf2 mediates the expression of HO-1 and NAD(P)H quinone oxidoreductase gene in astrocytes and prevented neuronal damage related to superoxide (80) (Table 3).

***Protective effects of ellagic acid against heavy metals toxicity***

*Aluminum*

Aluminum (Al) is easily accumulated in the brain, bone, liver, and kidney of mammalian tissues and increases the risk of health disorders (81). Administration of EA (60 mg/kg, PO) ameliorated hepatic dysfunction, dyslipidemia, hepatic histological alterations, reduced liver MDA and protein carbonyl content levels, and elevated liver CAT, GPx, and SOD activity and GSH content in Al toxicants (82). Furthermore, EA administration decreased serum level of total cholesterol, triglycerides, low-density lipoprotein cholesterol (LDL-C), and very-low-density lipoprotein cholesterol, and increased the high-density lipoprotein cholesterol (HDL-C) level in AlCl_3_ treated rats (82) (Table 4).

*Arsenic*

Arsenic (As) is a known toxic element that is present in the air, water, and soil. Based on the experimental studies, it has toxic effects on vital organs such as the kidney, brain, and liver (83, 84). The toxicity of As significantly enhanced the generation of free hydroxyl radicals, superoxide anions, dimethyl arsenic peroxy radical, dimethyl arsenic radical, and nitric oxide (85). Treating rats with EA (40 mg/kg, PO) during As exposure significantly decreased the inflammatory (IL-1β, TNF-α, and INF-γ) and apoptotic markers; EA attenuated MMP and ROS generation (86). Sodium arsenate (SA) in the rat brain increased oxidative stress elements (MDA, NO, and PC) level and inflammatory markers (IL-1β and TNF-α), and decreased the total antioxidant capacity, GPx, and GSH content; these symptoms were reversed following EA administration (30 mg/kg, PO) (87). EA administration also reduced the plasma cardiac markers levels (AST, CK-MB, LDH, and cTnI) in SA-treated rats. Moreover, the heart of rats treated with SA displayed an increase in MDA and NO levels with a reduction in GSH content and activity of CAT, SOD, and GPx that were reversed by EA (30 mg/kg, PO) administration (88). Moreover, SA treatment in rats significantly diminished white blood cells, red blood cells, hemoglobin, hematocrit, and platelets and increased mean corpuscular volume and mean corpuscular hemoglobin; these all were attenuated with EA therapy (88). 

Pretreatment of the normal mammalian V79 cell lines with EA (75 µM) before As exposure significantly diminished the generation of ROS and DNA fragmentation (89). In rats, SA noticeably reduced levels of serum testosterone, total antioxidant capacity, GSH level, and the activity of antioxidant enzymes (SOD, CAT, and GPx) in testicular tissue which increased with EA (30 mg/kg, PO) supplementation. Furthermore, they showed SA enhanced the levels of MDA, IL-1β, TNF-α, and NO that were suppressed by EA (90). 

Overall, EA due to antioxidant and anti-inflammatory properties protected the body against As toxicity (Table 4).

*Arsenic trioxide*

Arsenic trioxide (As_2_O_3_) is an effective drug in treating acute promyelocytic leukemia due to its substantial anticancer effect (91). As_2_O_3 _could cause cardiotoxicity, QTc prolongation, torsades de pointes, endothelial dysfunction, and sudden death (91). As_2_O_3 _treated SH-SY5Y human neuroblastoma cell lines significantly indicated a decrease in cell viability via increasing MMP and releasing cytochrome c and inducing DNA damages via generation of ROS which was reversed with EA (20 µM) pretreatment (92). EA (30 mg/kg, PO) protected against As_2_O_3 _(5 mg/kg, daily, for 10 days, IP) cardiac toxicity. In this study, EA decreased MDA content and increased GSH and GPx activity, and diminished QTc interval prolongation (93) (Table 4).

*Iron*

Iron is an important nutrient that is crucial for various functions such as oxygen transport, cell respiration, and DNA synthesis; however, extra iron accumulation in the liver leads to the generation of oxidative stress and hepatotoxicity (94). Iron overload in mice increased liver iron content, ROS, and levels of ALP, ALT, AST, and LDH. It decreased antioxidant enzyme activity (SOD, GST, and CAT), and induced liver damage, fibrosis, and hepatotoxicity (95). EA (8 mg/kg, PO) supplementation prevented iron overload toxicity and reversed these changes in the mice’s liver tissue. Furthermore, EA protected the liver of mice against iron overload-induced apoptosis by a decrease in the Bax/Bcl-2 ratio and inhibiting caspase-3 activation (95) (Table 4).

***Protective effect of ellagic acid against synthetic toxicants***

*6-Hydroxydopamine*

6-Hydroxydopamine (6-OHDA) is a neurotoxic agent and plays an important role in preclinical research to induce Parkinson’s disease model. 6-OHDA is uptaken by dopamine or noradrenaline membrane transporters due to its structural similarity with endogenous catecholamines (96). 6-OHDA causes neuronal damage by the generation of oxidative stress and ROS formation (96). EA (50 mg/kg, PO) supplementation reduced 6-OHDA brain toxicity. It decreased MDA, ROS, and DNA fragmentation. Also, this study demonstrated the protective effect of EA against 6-OHDA via activation of the ERβ/Nrf2/HO-1 cascade (97). Moreover, Farbood Y *et al*. study showed that injection of 6-OHDA into the right medial forebrain bundle in rats increased levels of TNF-α and IL-1β, which ameliorated with EA (50 mg/kg, PO) treatment (98) (Table 5).

*Acrylamide*

Acrylamide is considered a neurotoxic agent (99). Treating rats with acrylamide increased the generation of MDA, NO, Il-β, and TNF-α and decreased GSH, SOD, GPx, and CAT in the brain tissue; co-treatment with EA (30 mg/kg, PO) prevented these changes (100). Also, acrylamide exposure causes neuron and glial cell necrosis in the brain cortex and EA provided a protective effect against them (Table 5).

*Carbon tetrachloride*

Carbon tetrachloride (CCl_4_), a known hepatotoxic agent, has been used as an effective solvent and cleaning agent in industrial manufacturers (101). Exposure to CCl_4_ leads to liver injury and increases levels of GDH, ALP, ALT, and AST (101). EA (10 mg/kg, IP) protected the rat liver against CCl_4_ toxicity (102). CCl_4_ exposure induces cirrhosis through the activation of ROS formation and angiogenesis by expressing vascular endothelial growth factor and vascular endothelial growth factor receptor 2, and increasing caspase-3 activity. These aforementioned effects were noticeably reversed by EA administration (103). EA (15 mg/kg, PO) supplementation also effectively diminished the plasma levels of ALT, AST, and albumin, and significantly suppressed the gene expression of iNOS and collagen I in cirrhosis mice (103) (Table 5).

*Dichloroacetate*

Dichloroacetate (DCA) is mainly formed during the chlorination process of drinking water. It has been shown that DCA has toxic effects on various organs and can induce liver cancer (104). DCA caused developmental abnormalities and significantly enhanced levels of superoxide anion and NO in zebrafish embryos. EA (20 mM) protected against these changes (105) (Table 5).

*2,3,7,8-Tetrachlorodibenzo-p-dioxin*

2,3,7,8-Tetrachlorodibenzo-*p*-dioxin (TCDD) formed as an unwanted product in waste burning or as a side product in organic synthesis, is one of the most toxic and representative compounds of dioxins and its toxicity is mediated by the aryl hydrocarbon receptor (106). TCDD administration to rats meaningfully produced superoxide anion, LPO, and DNA single-strand breaks in the brain, and EA treatment (1 mg/kg, PO) reversed these changes (107). In the rat brain, EA (1 mg/kg, PO) significantly increased SOD and CAT activities (108). EA (100 mg/kg, PO) supplementation also protected against TCDD-induced nephrotoxicity in rats by improving antioxidant enzymes activity (SOD, GSH, GPx, and CAT), CYP1A1, and ATPase enzymes activity (Na^+^/K^+^-ATPase, and Mg^2+^-ATPase) with a significant decrease in Ca^2+^-ATPases activity (109). The membrane-bound enzymes such as Na^+^/K^+^-ATPase, Mg^2+^-ATPase, and Ca^2+^-ATPase are responsible for the transport of sodium/potassium, magnesium and calcium ions by inhibition of which TCDD causes nephrotoxicity (109). TCDD (100 ng/kg daily for 8 weeks, PO) induced testicular toxicity, decreased sperm concentration and motility, and the testis levels of SOD, CAT, and GSH. Also, it increased testicular MDA and LPO levels. EA (2 mg/kg daily for 8 weeks, PO) administration reversed all these defects (110). Moreover, EA supplementation protected testicular tissue against TCDD-induced histopathological changes such as degeneration, desquamation, disorganization, reduction in germinal cells, interstitial edema, and congestion (110) (Table 5).

***Metabolism, safety, and dose translation to humans***

Unlike food containing EA, free EA is mainly absorbed in the stomach and can be detected in the plasma 2 hours after intake. Urolithin derivatives are the main EA-derived metabolites that are produced by human intestinal microbiota, and after metabolizing phase 1 and 2, urolithin A and B conjugates are the main detectable forms in plasma, urine, and some tissues in humans. Enterohepatic circulation contributes to their long-time persistence in plasma and urine (up to 48-72 hr) (3, 111). However, individual variability in response to dietary EA due to different types of metabotypes associated with different types of gut microbiota should be taken into account (112).

No adverse effect have been reported in humans with EA when this compound was consumed as a nutrition supplement or as a part of the diet (3). However, based on animal studies, a high portion of ellagitannins, as a complex form of EA, induces antinutritional effects because they inhibit β-galactosidase and bind with some dietary proteins, fibers, and minerals that may result in malabsorption (113). The toxicological effects of this compound have not been thoroughly investigated. The LD_50_ (lethal dose for 50% of the population) of EA in the rat after IP administration has been reported to be 630 mg/kg. In a 90-day sub-chronic study, EA caused no observable changes in male rats, while a slight reduction in body weight of female rats was observed. Based on this study, the no-observed-effect level (NOEL) was estimated to be 3011 mg/kg b.w./day for males and less than 778 mg/kg b.w./day for female rats. Meanwhile, the no-observed-adverse-effect level (NOAEL) was estimated at 3254 mg/kg b.w./day for female rats (114). 

Dose translation from animals to humans is based on an indirect formulation which is normalized by body surface area (BSA): Human equivalent dose (mg/kg) = Animal Dose (mg/kg)×(animal K_m_/human K_m_), where K_m_ value results from body weight (kg) divided by BSA (m^2^). Therefore, 50 mg/kg in mice whose weight and BSA are respectively 0.02 kg and 0.007 m^2^ means about 4 mg/kg for a 60 kg adult human with 1.6 m^2^ BSA (115). 

In this regard, for starting a clinical trial in healthy adult volunteers based on animal studies, the human equivalent dose is calculated using the BSA normalization of the safe animal dose (115). 

**Table 1:** Summarized data of EA protection against synthetic drugs’ adverse effects or toxicity

***Toxic agent***	***Dose/Concentration period and route of exposure***	***Dose/Concentration of EA treatment period and route of administration***	***In vivo***	***Results***	***Reference***
**CPM**	200 mg/kg, Single-dose, *IP*	10 mg/kg for 5 days, *IP*	Male Wistar rats	Decreased MDA, AOPP, and XO levels and increased the activities of GSH and CAT. Diminished ROS, AST, ALT, ALP, and GGT in the liver.	(18)
	15 mg kg, once a week, for 8 weeks, *p.o*	2 mg/kg every day, for 8 weeks, *p.o*	Male Sprague-Dawley rats	Ameliorated epididymal sperm concentration and motility, and decreased MDA level and oxidative stress. Increased Bcl-2 and reduced Bax in testicular tissue.	(19)
	15 mg kg, once a week, for 8 weeks, *p.o*	2 mg/kg, every day for 8 weeks, *p.o*	Male Sprague-Dawley rats	Improved the tail and total abnormality of sperm, plasma MDA level, erythrocyte SOD activity, and erythrocyte CAT activity in testicular tissue.	(20)
	50 mg/kg, single dose, *IP*	100 mg/kg, daily, for 7 days, *p.o*	Male Swiss albino mice	Decreased BUN, Cr, LDH MDA, and XO levels and increased GSH content, GPx, and CAT activities in the kidney.	(21)
	150 mg/kg, single dose, *IP*	15 mg/kg, daily, for 14 days, *p.o*	Male Wistar rats	Attenuated LPO, MPO, and NO production and protein carbonyl level. Increased GPx and reduced ROS generation in the lung.	(22)
**CsA**	15 mg/kg, daily, for 21 days, *IP*	10 mg/kg, daily, for 21 days, *p.o*	Male Sprague–Dawley rats	Ameliorated the weights of testes and ventral prostate, epididymal sperm concentration, motility, increased testicular tissue GSH and CAT, and decreased MDA levels.	(24)
	15 mg/kg, daily, for 21 days, *s.c*	10 mg/kg, daily, for 21 days, *p.o*	Male Sprague–Dawley rats	Significantly increased the GSH level, and CAT activities, and decreased MDA level of kidney, liver, and heart tissues.	(25)
	15 mg/kg, daily, for 30 days, *p.o*	10 mg/kg, daily, for 30 days, *s.c*	Male albino rats	Reduced oxidative stress, LPO, and MDA levels and elevated CAT and Px activities and testicular GSH concentration.	(26)
	25 mg/kg, daily, for 21 days, *p.o*	50 mg/kg, daily, for 21 days, *p.o*	Male albino Wistar rats	Decreased the activities of serum hepatic enzymes such as AST, ALT, ALP and LDH, and LPO.	(27)
**Dox**	5 mg/kg, twice a week, for 2 weeks, *IP*	10 mg/kg, daily, for 2 weeks, *p.o*	Male Sprague–Dawley rats	Improved testicular relative weight, sperm count, motility, serum testosterone, testicular glycogen. Decreased oxidative stress, and TNF-α.	(29)
	20 mg/kg, single dose, *IP*	1 g mixed with diet and given for 8 weeks, *p.o*	Male C57BL/6 mice	Reduced ROS, IL-6, IL-10 levels, MDA and XO activities, and monocyte chemoattractant protein-1 and TNF-α levels in the cardia.	(30)
	5 mg/kg, twice a week, for 2 weeks, *IP*	10 mg/kg, daily, for 14 days, *p.o*	Male Sprague–Dawley rats	Decreased MDA, TNF-𝛼, iNOS, caspase-3, and cholinesterase and increased GSH levels in the brain.	(31)
**Gentamicin**	100 mg/kg, single dose, *IP*	10 mg/kg, daily for 10 days, *p.o*	Male Sprague Dawley rats	Decreased LPO and MDA and increased CAT, SOD enzyme activity, and GSH content in kidney tissue. Increased the ratio of Bcl-2/Bax and prevented MMP loss in the kidney.	(33)
**ISP**	100 mg/kg, daily, for 2 days, *s.c*	15 mg/kg, daily, for 10 days, *p.o*	Male Wistar rats	Suppressed troponin-I, creatine kinase, LDH, C-reactive protein, and TBARS. Increased SOD, GST, and CAT and restored the GSH content in heart tissue.	(36)
**MTX**	20 mg/kg, single dose, *IP*	10 mg/kg, daily, for 10 days, *p.o*	Male Wistar rats	Decreased MDA levels, Bcl-2/Bax ratio, cytochrome-c release, and caspase-3/9 and decreased mitochondrial outer membrane potential. Up-regulation of both Nrf2 and HO-1in the liver.	(38)
	20 mg/kg, single dose, *IP*	10 mg/kg, daily, for 5 days, *p.o*	Male Wistar rats	Reduced MDA, NO, and PGE2 levels and diminished the activity of MPO and XO, and increased GSH content level in the intestinal tissue.	(39)
	20 mg/kg, single dose, *IP*	10 mg/kg, daily, for 10 days, *p.o*	Male Wistar rats	Reduced AST, ALT, ALP, MDA, and LPO. Increased SOD, CAT, and GST, and restored the GSH content level in the liver.	(40)
**Paracetamol**	500 mg/kg, single dose, *p.o*	100 mg/kg, daily, for 7 days, *p.o*	Swiss albino mice	Suppressed liver marker enzymes (ALT, AST, and ALP) in serum and MDA levels. Increased CAT and SOD activity and GSH level in the liver.	(42)
**Sodium valproate**	400 mg/kg, daily for 7 days, *p.o*	50 mg/kg, daily, for 10 days, *p.o*	Male Wistar rats	Protected the male rats' reproductive system against necrosis, atrophy in seminiferous tubules, multi-nucleated giant cell formation, and interstitial edema induced by sodium valproate.	(44)
**STZ**	3 mg/kg single dose, ICV	35 mg/kg, daily, for 4 weeks, *p.o*	Wistar rats	Decreased the TBARS and LDH content and depression of GSH levels and SOD and CAT activity in the brain of rats. Decreased AChE and LDH activity, TNF-α, and eNOS levels.	(46)
	50 mg/kg, single dose, *IP*	50 mg/kg, daily, for 21 days, *p.o*	Wistar albino rats	Reduced MDA, TOS, and NO levels Increased TAS level, CAT, and PON-1 activities in sciatic and brain tissue.	(47)

AChE: acetylcholinesterase; AOPP: advanced oxidized proteins products; ALP: alkaline phosphatase; ALT: alanine aminotransferase; AST: aspartate aminotransferase; Bax: Bcl-2-associated X protein; Bcl-2: B-cell lymphoma 2; BUN: blood urea nitrogen; CAT: catalase; CPM: Cyclophosphamide; Cr: creatinine; CsA: Cyclosporine A; EA: ellagic acid; Dox: doxorubicin; eNOS: endothelial nitric oxide synthetase; GSH: Glutathione; GST: glutathione-S-transferase; GPx: glutathione peroxidase; GGT: gamma-glutamyl transferase; ICV: intracerebroventricular; IL-6 or 10: interleukin-6/-10; ISP: isoproterenol; IP: intraperitoneal; iNOS: inducible nitric oxide synthase; LPO: lipid peroxidation; LDH: lactate dehydrogenase; MTX: Methotrexate; MDA: malondialdehyde; MPO: myeloperoxidase; MMP: mitochondrial membrane potential; NO: nitric oxide; Nrf2: nuclear erythroid 2-related factor 2; Px: peroxidase; PGE2: prostaglandin-E2; PON-1: paraoxonase; p.o: oral; ROS: reactive oxygen species; STZ: streptozotocin; SOD: superoxide dismutase; TBARS: thiobarbituric acid reactive substances; TNF-α: tumor necrosis factor-alpha; TAS: total antioxidant status; TOS: total oxidant status; XO: xanthine oxidase

**Table 2:** Summarized data of EA protection against natural toxins

***Toxic agent***	***Dose/Concentration period and route of exposure***	***Dose/Concentration of EA treatment period and route of administration***	***In vitro/In vivo***	***Results***	***Reference***
**AFB1**	4 mg/kg, single dose, *IP*	300 mg/kg, two doses, *IP*	Male albino Wistar rats	Prevented the genotoxicity of AFB1 in the bone marrow.	(50)
**ConA**	20 mg/kg, single dose, *i.v*	200 mg/kg, single dose, *p.o*	Male BALB/c mice	Decreased the expression of TLR2 and TLR4 mRNA and proteins in the liver. Decreased NF-κB and expression of proinflammatory cytokines such as TNF-α, IL-6, and IL-1β.	(53)
**Glutamate**	60 mg/kg, daily, for 30 days, *p.o*	20 mg/kg, daily, for 30 days, *p.o*	Male albino rats	Increased SOD, GPx, GRx, and CAT and diminished MPO and XO. Elevated testosterone hormone levels enhanced male reproductive capacity, and inhibited histological and ultrastructure testicular damage.	(57)
**LPS**	50 μg/kg, single dose, *IP*	20 mg/kg, single dose, 1 hr before exposure	Male BALB/c mice	Reduced hepatic MDA content, TNF-α, and serum ALT and AST levels. increased expression of Nrf2 and HO-1.	(60)
**Ethanol**	7.9 g/kg, daily for 45 days, *p.o*	90 mg/kg, daily for 45 days, *p.o*	Female albino rats	Decreased TBARS, hydroperoxides, NO, ALT, AST, LHD, and ALP, and enhanced GSH level, SOD, and GST.	(65)
	7.9 g/kg, daily for 45 days, *p.o*	90 mg/kg, daily for 45 days, *p.o*	Female albino Wistar rats	Improved body weight, restoring antioxidant status, modulating micronutrients, and attenuating lipid levels. Reduced AST, ALT, and ALP.	(65)
	100 mM for 24 hr	100 μM, before exposure	hepatic HepG2 cells line	Reduced NO, TGF-β1, and SR-B1.	(66)
**Nicotine**	5 mg/kg, daily for 15 days, *IP*	60 mg/kg, daily for 15 days, *IP*	Sprague-Dawley rats	Increased body weight, total GSH, GPx, and SOD activities, and decreased MDA and NO levels in the kidney	(69)

AFB1: Aflatoxin B1; ALT: alanine aminotransferase; AST: aspartate aminotransferase; CAT: catalase; ConA: concanavalin A; EA: ellagic acid; GRx: Glutaredoxins; GPx: glutathione peroxidase; IL-6: interleukin-6; IL-1β; interleukin-1β; IP: intraperitoneal, i.v: intravenous; LPS: Lipopolysaccharide; MDA: malondialdehyde; MPO: myeloperoxidase; NF-κB: nuclear factor kappa-B; Nrf2: nuclear erythroid 2-related factor 2; p.o: oral; SOD: superoxide dismutase; TLR: toll-like receptor; TNF-α: tumor necrosis factor-alpha; XO: xanthine oxidase

**Table 3:** Summarized data of EA protection against toxicity of pesticides

***Toxic agent***	***Dose/Concentration period and route of exposure***	***Dose/Concentration of EA treatment period and route of administration***	***In vitro/In vivo***	***Results***	***Reference***
**Malathion **	0.5 mg/L for 14 days, dissolved in water	Diet contains EA in aqua solution	*Cyprinus carpio* (fish)	Diminished MDA and increased SOD, CAT, and GSH-Px, activities in the liver and kidney.	(72)
**Paraquat**	45 mg/kg single dose, *IP*	85 mg/kg, 1 hr after exposure, *p.o*	Female albino Wistar rats	Improved kidney tissue structure histologically and increased TAS and TOS.	(74)
	100 *μ*M for 72 hr	80 μM for 72 hr	Human lung carcinoma, A549 cells	Decreased ROS, LPO, and LDH and up-regulated Nrf2, HO-1, and NQO1.	(75)
**Phosalone**	0.11 mM for 24 hr	100 nM for 24 hr	Rat embryonic fibroblast	Suppressed ROS and LPO and inflammatory cytokines (TNF-α, IL-1β, IL-6, and NF-κB).	(77)
**Rotenone**	1 mg/kg, daily for 5 weeks, *s.c*	100 mg/kg, daily for 5 weeks, *p.o*	Wild-type C57BL/6J male mice	Activated the Nrf2/HO-1 signaling pathways in the brain.	(80)

CAT: catalase; EA: ellagic acid; GST: glutathione-S-transferase; GSH-Px: Glutathione peroxidase; IL-6: interleukin-6; IL-1β: interleukin-1β; IP: intraperitoneal; LPO: lipid peroxidation; LDH: lactate dehydrogenase; MDA: malondialdehyde; NF-κB: nuclear factor kappa-B; NQO1: NAD(P)H quinone oxidoreductase; Nrf2: nuclear erythroid 2-related factor 2; p.o: oral; ROS: reactive oxygen species; s.c: subcutaneous; SOD: superoxide dismutase; TAS: total antioxidant status; TNF-α: tumor necrosis factor-alpha; TOS: total oxidant status

**Table 4:** Summarized data of EA protection against heavy metals

***Toxic agent***	***Dose/Concentration period and route of exposure***	***Dose/Concentration of EA treatment period and route of administration***	***In vitro/In vivo***	***Results***	***Reference***
**Aluminum**	20 mg/kg, daily, for 8 weeks, *p.o*	60 mg/kg, daily for 8 weeks, *p.o*	Male albino rats	Reduced liver MDA levels and modulated elevation of liver CAT, GPx, and SOD activity and GSH level.	(82)
**Arsenic**	10 mg/kg, daily, for 8 days, *p.o*	40 mg/kg, daily, for 11 days, *p.o*	Wistar rats	Decreased the inflammatory markers (IL-1β, TNF-α, and INF-γ) and apoptotic markers in the brain, down-regulated Bax and up-regulated Bcl-2	(86)
	10 mg/kg, daily, for 21 days, *p.o*	30 mg/kg, daily, for 14 days, *p.o*	Male Wistar albino rats	Diminished MDA and NO levels and inflammation markers (IL-1β and TNF-α), and increased TAC, GPx, and GSH levels in the brain.	(87)
	10 mg/kg, daily, for 21 days, *p.o*	30 mg/kg, daily, for 14 days, *p.o*	Male Wistar rats	Decreased AST, CK-MB, LDH, and cTnI; and decreased MDA and NO with the increase of GSH level and activities of CAT, SOD, and GPx in the cardia.	(88)
	500 µM for 24 hr	75 µM, 1 hr prior to exposure	Chinese hamster V79 cells	Diminished the generation of ROS and DNA fragmentation.	(89)
	10 mg/kg daily for 21 days, *p.o*	30 mg/kg daily for 14 days, *p.o*	Male Wistar rats	Increased TAC, GSH level, and the activity of antioxidant enzymes (SOD, CAT, and GPx) in testicular tissue. Suppressed MDA, IL-1β, TNF-α, and NO.	(90)
**Arsenic trioxide**	2 µM for 24 hr	20 µM, 1 hr prior to exposure for 24 hr	SH-SY5Y human neuroblastoma cell line	Attenuated MMP loss and released cytochrome c and DNA damage.	(92)
	5 mg/kg, daily, for 10 days, *IP*	30 mg/kg, daily, for 10 days, *p.o*	Male Wistar rats	Decreased MDA and increased GSH, and GPx activity, and attenuated QTc prolongation in the heart.	(93)
**Iron**	100 mg/kg, for 5 alternative days, *IP*	8 mg/kg, daily, for 21 days, *p.o*	Swiss albino male mice	Suppressed ROS generation and serum marker levels (ALP, ALT, AST, and LDH) and increased antioxidant enzymes (SOD and CAT). Decreased the Bax/Bcl-2 ratio and inhibited the caspase-3 activation.	(95)

ALP: alkaline phosphatase; ALT: alanine aminotransferase; AST: aspartate aminotransferase; Bax: Bcl-2-associated X protein; Bcl-2: B-cell lymphoma 2; CAT: catalase; CK-MB: Creatine kinase-MB; GPx: glutathione peroxidase; GSH: Glutathione; IL-1β; interleukin-1β; INF-γ: Interferon-gamma; LDH: lactate dehydrogenase; MDA: malondialdehyde; MMP: mitochondrial membrane potential; NO; nitric oxide; p.o: oral; ROS: reactive oxygen species; SOD: superoxide dismutase; TAC: total antioxidant capacity; TNF-α: tumor necrosis factor-alpha

**Table 5:** Summarized data of EA protection against synthetic toxicants

**Toxic agent**	**Dose/Concentration period and route of exposure**	**Dose/Concentration of EA treatment period and route of administration**	***In vitro*** **/** ***In vivo***	**Results**	**Reference**
**6-OHDA**	5 μl microinjection into the left striatum	50 mg/kg, daily for 1 week, *p.o*	Male Wistar rats	Decreased MDA and ROS formation, and DNA fragmentation activated ERβ/Nrf2/HO-1 cascade	(97)
	16 μg/2 μl injection into the right MFB	50 mg/kg, daily for 10 days, *p.o*	Male Wistar rats	Decreased the levels of TNF-α and IL-1β in MFB.	(98)
**Acrylamide**	20 mg/kg, daily for 30 days, *p.o*	30 mg/kg, daily for 30 days, *p.o*	Male Wistar rats	Diminished MDA, NO, Il-β, and TNF-α, and enhanced GSH, SOD, GPx, and CAT in brain tissue.	(100)
**CCl**_4_	1.5 ml/kg, twice a week for 4 weeks, *IP*	10 mg/kg, 5 times a week for 8 weeks, *IP*	Male Wistar albino rats	Decreased Bcl-2 and NF-kB expression and the MDA level. Up-regulated caspase-3 and Nrf-2	(102)
	2 ml/kg, 3 times a week for 6 weeks, *IP*	15 mg/kg, daily for 5 weeks, *p.o*	C57BL/6 male mice	Decreased ROS formation and angiogenesis by VEGF and VEGFR2, and the caspase‑3 activity. Decreased serum levels of ALT, AST, and albumin.	(103)
**Dichloroacetate**	32 mM, incubated for 144 hr	20 mM, incubated for 144 hr	Zebrafish	Suppressed the developmental abnormalities and significantly reduced levels of NO in zebrafish embryos.	(105)
**TCDD**	46 ng/kg, daily for 90 days, *p.o*	1 mg/kg, daily for 90 days, *p.o*	Female Sprague-Dawley rats	Diminished LPO and DNA SSBs in the brain.	(107)
	46 ng/kg, daily for 90 days, *p.o*	1 mg/kg, daily for 90 days, *p.o*	Female Sprague-Dawley rats	Significantly increased SOD, CAT, and GPx activities, as well as GSH levels.	(108)
	15 µg/kg for 21 days, *p.o*	10 mg/kg daily for 21 days, *p.o*	Male albino Wistar rats	Attenuated antioxidant enzyme activities (SOD, GSH, GPx, and CAT), and CYP1A1 and ATPase enzyme activities.	(109)
	100 ng/kg daily for 8 weeks, *p.o*	2 mg/kg daily for 8 weeks, *p.o*	Male Sprague-Dawley rats	Enhanced sperm motility and concentration, SOD and CAT activity, and GSH content level, and increased MDA and LPO levels.	(110)

6-OHDA: 6-hydroxydopamine; ALP: alkaline phosphatase; ALT: alanine aminotransferase; AST: aspartate aminotransferase; Bcl-2: B-cell lymphoma 2; CAT: catalase; CCl4: Carbon tetrachloride; ERβ: Estrogen receptor beta; GSH: Glutathione; GST: glutathione-S-transferase; GPx: glutathione peroxidase; IL-1β; interleukin-1β; IP: intraperitoneal; LPO: lipid peroxidation; LDH: lactate dehydrogenase; MDA: malondialdehyde; MFB: mid frontal brain; NF-κB: nuclear factor kappa-B; NO; nitric oxide; Nrf2: nuclear erythroid 2-related factor 2; p.o: oral; ROS: reactive oxygen species; SOD: superoxide dismutase; SR-B1: Scavenger receptor class B type 1; SSBs: single-strand breaks; TGF-β1: Transforming growth factor-beta; TNF-α: tumor necrosis factor-alpha; TCDD: 2,3,7,8-tetrachlorodibenzo-p-dioxin; TBARS: thiobarbituric acid reactive substances; VEGF: vascular endothelial growth factor; VEGFR2: vascular endothelial growth factor receptor 2

**Figure 3:**
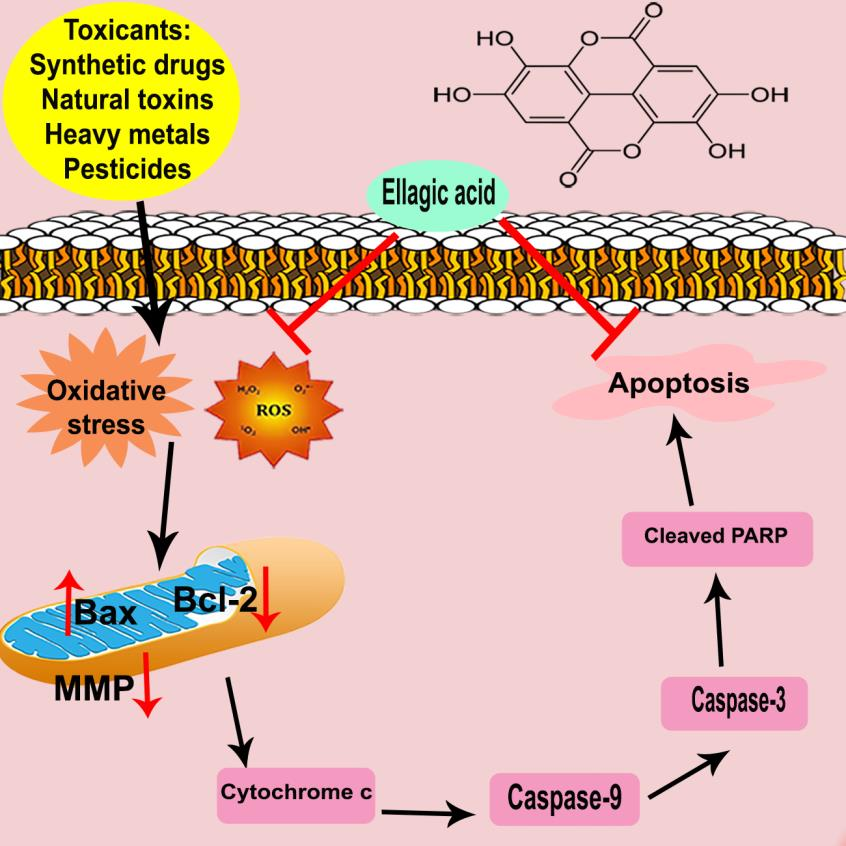
Schematic diagram of ellagic acid anti-apoptotic effects against chemical and natural toxins

Bax: Bcl-2-associated X protein; Bcl-2: B-cell lymphoma 2; MMP: mitochondrial membrane potential; ROS: reactive oxygen species

## Conclusion

Taken together, several available studies on EA indicate the protective effects of this natural product against the adverse effects of many toxins or toxic agents. 

In this review, we summarized findings implicate the antidotal/protective effects of EA against some types of chemical drugs, natural toxins, pesticides, heavy metals, and synthetic compounds *in vivo* and *in vitro*. Based on the gathered data, the ameliorative effects of EA are via several mechanisms including (i) activation of the antioxidant response through the Nrf2/HO-1 signaling pathway; (ii) inhibition of pro-inflammatory agents, such as iNOS, COX-2, and cytokines by inhibiting nuclear factor-kappa B (NF-ĸB); (iii) alteration of several growth factors expression, as transforming growth factor-beta (TGF-α), vascular endothelial growth factor, and vascular endothelial growth factor receptor; (iv) modulation of several cell survival and cell-cycle genes such Bcl-2, Bax, caspase-3 and tumor suppressors (p53); and (v) regulation of kinases, like phosphoinositide 3-kinase (PI3-K) and GSK-3β. Also, it protects against DNA damage and provides an extracellular complex with some toxins, and may reduce their interaction with cells (Figure 3).

Altogether, it needs more work, in particular, randomized clinical trials, to confirm the effects and also consider any probable unwanted effects. To the best of our knowledge, no clinical trial has been done in this area and further research should be done to reveal more about these mechanisms in humans due to linking the bench to the bed, the ultimate goal of researchers.

## Authors’ Contributions

KN and MR wrote the manuscript; HH designed the study; All authors read and approved the final manuscript.

## Conflicts of Interest

The authors declare that there are no conflicts of interest. 
